# Micronutrient intake in adults with drug-resistant epilepsy treated with the modified Atkins diet needs monitoring

**DOI:** 10.3389/fnut.2026.1797011

**Published:** 2026-05-07

**Authors:** Ida Kjendbakke, Kaja Kristine Selmer, Karl Otto Nakken, Dag Hofoss, Per Ole Iversen, Magnhild Kverneland

**Affiliations:** 1 National Centre for Epilepsy, Division of Clinical Neuroscience, Oslo University Hospital, Oslo, Norway; 2 Department of Research and Innovation, Division of Clinical Neuroscience, Oslo University Hospital, Oslo, Norway; 3 Department of Nutrition, University of Oslo, Oslo, Norway; 4 Department of Haematology, Oslo University Hospital, Oslo, Norway

**Keywords:** epilepsy, ketogenic diet, mineral, modified Atkins diet, nutrient, vitamin, electrolyte, potassium

## Abstract

The ketogenic diet, a high-fat, low-carbohydrate diet, is an established treatment for patients with severe epilepsy. We have previously reported a moderate reduction in seizure frequency in drug-resistant epilepsy after treatment with a modified Atkins diet (MAD). In this study, we report the mean dietary intake, including micronutrients, in 56 adults, prospectively recorded at 4 and 12 weeks. All reported values describe food intake alone, excluding vitamin and mineral supplements. To the best of our knowledge, this is the first report of micronutrient intake in adults on the MAD. The mean dietary intake of fat-soluble vitamins A, D, and E was higher, while the intake of several water-soluble vitamins, electrolytes, and minerals was lower compared with that of a population-based reference population consuming a habitual diet. This difference was most likely due, in line with expectations, to the high-fat, low-carbohydrate dietary content. Dietary intake of vitamin C and potassium in both men and women was 25–50% lower than that recommended by the Nordic Nutrition Recommendations 2023 (NNR 2023). Among women, vitamin D, folate, calcium, iodine, and selenium were also 25–50% lower than recommended, while iron at the 12-week follow-up was >50% lower. Furthermore, magnesium and zinc were lower than the recommended levels but less pronounced (<25%) in both men and women. Among women, riboflavin and vitamin B6 were <25% below the recommended levels, and among men, folate, calcium, and selenium were <25% below recommendations. The mean fiber intake of the study population (24 g/day) was in line with the reference population and almost in accordance with recommendations. When combining intake from diet and supplements, the mean intake of each nutrient was within the NNR 2023 recommendations. Our findings indicate that treatment with MAD resulted in lower dietary intakes of several water-soluble vitamins, electrolytes, and minerals compared with both a reference population and the NNR 2023 recommendations, highlighting the need for careful dietary monitoring, advice, and supplementation during MAD treatment.

## 1 Introduction

The ketogenic diet is a high-fat, very-low-carbohydrate diet that dampens epileptic seizures, and such dietary treatment has been used in epilepsy treatment for more than a century (1).

Variants of low-carbohydrate, high-fat diets are also used for weight reduction and diabetes (2), and in the last decade, research has been carried out to apply ketogenic dietary treatment to other neurologic and psychiatric disorders such as migraine, Parkinson’s disease, Alzheimer’s disease, bipolar disorder, and depression (3–6). Although the mechanisms are incompletely understood (7), it is well established that such a diet, mimicking the metabolic changes of fasting, has beneficial health effects.

Adults who experience a reduction in seizure frequency or other beneficial effects may continue such dietary treatment for many years, even lifelong. Ketogenic diets represent a radical dietary change in macronutrients. This change may also imply a risk of vitamin and mineral deficiency or excess. Guidelines on ketogenic dietary treatment of drug-resistant epilepsy agree that dietary supplements are necessary (8, 9). However, data on how a ketogenic diet affects micronutrients in adults are scarce (8, 9).

Despite its limitations, a modified Atkins diet (MAD) offers the possibility of a more easily implemented and potentially healthier dietary pattern. Choosing plant oils high in monounsaturated and polyunsaturated fatty acids instead of animal fats promotes cardiovascular health. Even with a limit of 20 g of carbohydrate (sugars) per day, there is room for some vegetables. Fish is also a natural part of a ketogenic diet, along with fatty dairy products such as cheese and cream. Substituting flour, rice, potatoes, and other starches with nuts, seeds, and some vegetables provides high-fiber, heart-healthy alternatives. Moreover, all of these choices greatly impact the micronutrient content of a ketogenic diet.

Our study aimed to investigate micronutrient intake among adults with drug-resistant epilepsy treated with a MAD and to evaluate whether the patients comply with the recommendations of the Nordic Nutrition Recommendations 2023 (NNR 2023) (10). To the best of our knowledge, this is the first report on micronutrient intake in adults on MAD.

## 2 Materials and methods

This prospective study involved 56 adults (30 women) with drug-resistant epilepsy treated with the MAD for 12 weeks. Dietary intake was recorded after 4 and 12 weeks (11). A detailed description of the study population, study design, baseline patient characteristics, and dietary intervention is provided in the supplementary document.

### 2.1 Pre-keto diet

In Norway, it is common to eat sandwiches for breakfast, lunch, and the evening meal. Bread is wheat- or rye-based, containing 25–75% whole meal and is usually served a thin layer of margarine or butter. Common toppings are meat, fish, cheese, eggs, nut spreads, mayonnaise, salads, jam, fruits, and vegetables. Traditionally, dinner consists of meat or fish along with carbohydrate-rich staples such as potatoes, pasta, or rice, and fresh or boiled vegetables.

Among the participants, pre-keto habits with respect to desserts, sweets, cakes, chocolate, and various carbohydrate-rich snacks varied widely—from daily to never. For 40 years preceding this study, Norwegian health authorities advised limiting the intake of fat, in particular saturated and trans fats.

### 2.2 Diet assessment

Before starting the treatment, the patients were admitted for a short hospital stay, including education from a dietician (MK) on how to prepare for the diet. At the 4- and 12-week assessments, they submitted a 3-day dietary record in which they recorded their intake of foods and drinks, specified and weighed using a household scale with a precision of 1 g. The food records were completed within 2 weeks before the short hospital stay at 4- and 12-week follow-ups and were collected on 2 weekdays (Monday and Friday) and 1 weekend day (Saturday or Sunday) (12). Participants received proper training on how to weigh foods while preparing meals, and they borrowed high-accuracy scales for the 12-week project period. Nutrient intake was reported in grams of food eaten. Nutritional intake was based on two 3-day records and was analyzed using the Norwegian Food Composition Database (13). The ketogenic ratio is defined as the ratio between fat and protein + carbohydrate in grams. Adherence to the diet was ensured through the measurement of ketone levels in blood and urine, as described earlier (14).

### 2.3 Modified Atkins diet

Participants’ dietary intake of carbohydrates was limited to 16 g per day. As the Norwegian Food Composition Database (13) excludes fibers from carbohydrates, the carbohydrates were limited to those digestible by the small intestine, while fibers were eaten in free amounts. Participants were encouraged to consume high-fat foods, with emphasis on monounsaturated and polyunsaturated fatty acids. They were advised to eat adequate amounts of protein-rich foods. According to the international consensus guidelines, participants received multivitamin, multimineral, and calcium supplements (15, 16). They were advised to eat as many vegetables as possible within the limits of 16 g of carbohydrates per day.

The study participants were supplied with one multivitamin and multimineral tablet per day “Nycoplus Multi 11 vitamins and 8 minerals” containing vitamin A 500 μg retinol equivalents (RE), vitamin D 5 μg, vitamin E 10 μg, thiamine 1.4 mg, riboflavin 1.6 mg, niacin 18 mg (NE), vitamin B6 2 mg, vitamin B12 1 μg, folate 200 μg, vitamin C 60 mg, iron 14 mg, zinc 15 mg, copper 2 mg, iodine 150 μg, manganese 2.5 mg, chromium 50 μg, selenium 50 μg, magnesium 100 mg, and calcium carbonate (Nycoplus) powder 800 mg per day.

### 2.4 Outcome measures

At two time points, the collected dietary records were analyzed using the Norwegian Food Composition Table (13). We determined intake of energy in megajoules [MJ], saturated fatty acids (SFA), monounsaturated fatty acids (MUFA), and polyunsaturated fatty acids (PUFA), protein, carbohydrate, and fiber in grams and energy percentage [g and E%]. Moreover, dietary intake (excluding supplements) of the following micronutrients was calculated: vitamin A [μg RE], vitamin D [μg], vitamin E [μg], thiamine [mg], riboflavin [mg], vitamin B6 [mg], vitamin B12 [μg], folate [μg], vitamin C [mg], calcium [mg], magnesium [mg], sodium [g], potassium [g], phosphorous [g], iron [mg], zinc [mg], copper [mg], iodine [μg], and selenium [μg].

### 2.5 Reference population

We used data from the Norkost 4 study population as a reference population. Norkost 4 is a Norwegian survey from 2022 to 2023, collecting dietary intake of 915 men and 1,049 women aged 18 to 80 years (17, 18). For comparison, Norkost 4 values excluding dietary supplements were used.

### 2.6 Nordic nutrition recommendations 2023 (NNR 2023)

The NNR 2023 provides recommendations for intake of macronutrients and micronutrients as recommended intake (RI), adequate intake (AI), and upper intake level (UL) (10). RI is the average intake of nutrients that is sufficient for 95% of a population, while AI is the average daily intake level based on estimations of nutrient intake by a specified group. UL is the highest intake that does not cause negative health effects. NNR 2023 provides intake ranges for macronutrients given as a percentage of total energy intake (E%) (10).

### 2.7 Statistical analysis

Data are presented as frequencies or means (with ranges or 95% confidence intervals [CI]). The Shapiro–Wilk normality test and histograms were used to assess data distribution, showing that the majority of the data were normally distributed. A one-sample t-test was used to compare the dietary intake of micronutrients and macronutrients at 4 and 12 weeks on MAD, with the mean dietary intake in Norkost 4 (17). *p*-values of less than 0.05 were considered statistically significant for all tests. We used IBM SPSS Statistics.

## 3 Results

The mean age of the study participants was 37 years (16–65 years); 36 (61%) were women, and 22 (37%) had intellectual disabilities. Supplementary Figure S1 in the supplement provides an overview of the study population flow.

### 3.1 Macronutrients

Table 1 shows macronutrient intake in the study group after 4 and 12 weeks on MAD compared with the healthy reference population (Norkost 4) and the recommended intake (NNR 2023). The mean ketogenic ratio was 1.7 at both follow-ups.

**Table 1:** Estimated macronutrient intake at 4 and 12 weeks on the MAD for men and women combined, compared to a healthy reference population (Norkost 4) and recommended intake (NNR 2023).

	Norkost 4 *n* = 1964	4 weeks on MAD (SD) (*n* = 52)	Comparison of 4 weeks MAD to Norkost 4		12 weeks on MAD (SD) (*n* = 44–45)	Comparison of 12 weeks MAD to Norkost 4		NNR 2023
			Mean difference (SD)	*p*		Mean difference (SD)	*p*	
Energy, MJ	9.2 (3.8)	8.3 (2.6)	−0.9 (2.6)	0.004	8.3 (3.0)	−1.0 (2.9)	0.027	
Protein, g	94 (43)	88 (28)	−6 (28.2)	0.127	88 (36)	−6.0 (37.0)	0.280	
Protein, E%	18 (6)	18 (4)	0.2 (4.4)	0.736	18 (5)	0.40 (4.7)	0.570	10–20
Fat, g	94 (47)	171 (59)	75.9 (60.2)	<0.001	169 (68)	73.8 (69.0)	<0.001	
Fat, E%	38 (9)	77 (5)	38.7 (5.2)	<0.001	76 (6)	38.2 (5.9)	<0.001	25–40
SFA, g	34 (20)	53 (21)	19.0 (21.8)	<0.001	51 (26)	16.9 (26.6)	<0.001	
SFA E%	13 (5)	24 (5)	11.0 (5.5)	<0.001	23 (7)	10.0 (6.7)	<0.001	<10
MUFA, g	37 (20)	67 (29)	30.5 (29.5)	<0.001	66 (29)	29.3 (29.9)	<0.001	
MUFA, E%	15 (5)	30 (6)	15.1 (5.9)	<0.001	29 (6)	14.2 (5.8)	<0.001	10–20
PUFA, g	15 (9)	31 (13)	15.9 (13.7)	<0.001	30 (16)	15.1 (16.8)	<0.001	
PUFA, E%	6 (3)	14 (4)	7.9 (3.9)	<0.001	13 (4)	7.3 (4.6)	<0.001	5–10
Carbohydrates, g	219 (106)	14 (5)	−206.0 (5)	<0.001	14 (3)	−205 (3)	<0.001	
Carbohydrates, E%	40 (10)	3 (1)	−37.1 (1.4)	<0.001	3 (1)	−36.9 (1.2)	<0.001	45–60
Fiber, g	24 (12)	22 (11)	−2.2 (11.5)	0.164	24 (13)	−0.4 (13.4)	0.847	≥25–35
Fiber, E%	2 (0.9)	2 (1)	−0.2 (1.2)	0.298	2 (1)	0.4 (1.5)	0.102	

Values are presented as mean (SD) comparing values for baseline, 4 weeks, and 12 weeks of treatment using a one-sample t-test. Norkost 4 is the reference population. No supplements are included. SD=standard deviation; MAD=modified Atkins diet; SFA=saturated fatty acids; MUFA=monounsaturated fatty acids; PUFA=polyunsaturated fatty acids; NNR 2023=Nordic Nutrition Recommendations 2023; MJ=megajoules.

### 3.2 Micronutrients compared to the reference population

Details of micronutrient intake for men and women combined compared with Norkost 4 are provided in Table 2. Compared to Norkost 4 and corrected for energy intake, the mean intakes of thiamine, riboflavin, vitamin B6, vitamin C, calcium, iron, magnesium, sodium, potassium, and iodine were lower in women, men, or both in the study population. The mean intakes of fat-soluble vitamins A, D, and E were higher in the study population than in Norkost 4. In addition, the mean intake of selenium was higher in the study population than in the reference population. One participant was excluded from the vitamin A calculation because she had eaten 165 g of lamb liver containing 54,000 RE in one meal. Details about women and men are shown separately in Supplementary Tables S1A,B, respectively. Supplementary Table 2 presents the minimum and maximum intake of micronutrients in men and women in the study population.

**Table 2:** Estimated micronutrient dietary intake after 4 and 12 weeks on MAD for men and women combined, compared to the reference population (Norkost 4).

	Norkost 4 *n* = 1964	4 weeks MAD *n* = 52	Comparison of 4 weeks MAD to Norkost 4		Comparison of 12 weeks MAD to Norkost 4		NNR 2023	
	Mean (SD) / mean per 9.2 MJ	Mean (SD) / mean per 8.3 MJ	Mean difference (SD)	*p*	Mean difference (SD)	*p*	RI or AI men/ women >18 yrs	Upper limit
Vitamin A, μg RE	729 (821) / 79.2	868 (363)^1^/105	139 (366)	0.009	138 (464)	0.055	800/700	3,000
Vitamin D, μg	6 (7) / 0.7	10 (6) / 1.2	4 (6)	<0.001	4 (8)	0.002	10/10	100
Vitamin E, μg	15 (8) /1.6	27 (11) / 3.2	12 (11)	<0.001	11 (14)	<0.001	11/10	300
Thiamine, mg	1.7 (0.9) / 0.2	1.3 (0.5) / 0.16	−0.4 (0.6)	<0.001	−0.5 (0.6)	<0.001	0.1/0.1 /MJ	
Riboflavin, mg	2.0 (1.1) / 0.2	1.5 (0.6) / 0.18	−0.5 (0.6)	<0.001	−0.5 (0.8)	<0.001	1.6/1.6	
Vitamin B6, mg	1.8 (1.0) / 0.2	1.5 (0.5) / 0.18	−0.3 (0.6)	<0.001	−0.3 (0.7)	<0.001	1.8/1.6	25
Vitamin B12, μg	6.6 (6.1) / 0.7	7.7 (9.7) / 0.9	1.1 (9.9)	0.405	0.6 (9.2)	0.644	4/4	
Folate, μg	256 (124) / 27.8	257 (106) / 31.0	0.7 (108)	0.960	−6 (116)	0.735	330/330	1000^2^
Vitamin C, mg	97 (82) / 10.5	62 (36) / 7.5	−35 (37)	<0.001	−36 (43)	<0.001	110/95	1,000
Calcium, mg	969 (593) / 105.3	648 (327) / 78	−321 (335)	<0.001	−332 (485)	<0.001	950/950	2,500
Magnesium, mg	349 (150) / 37.9	291 (119) / 35.1	−58 (121)	<0.001	−77 (134)	<0.001	350/300	250^3^
Sodium, g	3.1 (1.7) / 0.3	2.1 (1.0) / 0.25	−1.0 (1.0)	<0.001	−1.1 (1.0)	<0.001	1.5/1.5	2.3
Potassium, g	3.7 (1.5) / 0.4	2.5 (0.8) / 0.30	−1.2 (0.8)	<0.001	−1.2 (1.0)	<0.001	3.5/3.5	
Phosphorous, g	1.7 (0.7) / 0.2	1.4 (0.5) / 0.17	−0.3 (0.5)	<0.001	−0.4 (0.6)	<0.001	0.52/0.52	3.0
Iron, mg	10 (5) / 1.1	9 (4) / 1.1	−1 (4)	0.017	−2 (5)	0.007	9/15	60
Zinc, mg	11.4 (5.9) /1.2	10.4 (3.9) / 1.2	−1.0 (4.0)	0.063	−1.8 (5.0)	0.016	12.7/9.7	25
Copper, mg	1.3 (0.6) / 0.1	1.2 (0.9) / 0.14	−0.1 (0.9)	0.569	−0.2 (0.9)	0.208	0.9/0.9	5
Iodine, μg	159 (203) / 17.3	119 (90) / 14.3	−40 (90)	0.002	−27 (118)	0.127	150/150	600
Selenium, μg	53 (42) / 5.8	68 (26) / 8.2	15 (26)	<0.001	13 (34)	0.015	90/75	255

Values are presented as mean (SD) using a one-sample t-test. Recommended intake is based on the NNR 2023. ^1^*n* = 51: one outlier removed. ^2,3^applies to intake from supplements. Norkost 4 is the reference population. No supplements are included. MAD=modified Atkins diet; SD=standard deviation; NNR 2023=Nordic Nutrition Recommendations 2023; MJ=megajoules; RE=Retinol Equivalents.

### 3.3 Micronutrients compared to recommendations

The mean intakes of vitamins A, D, E, thiamine, and B12 in the study population for both genders combined were in line with NNR 2023 (Table 3). The mean intakes of thiamine per MJ for men and women (not shown in any table) were 0.17/0.15 mg/MJ and 0.16/0.13 mg at 4 and 12 weeks, respectively, which is in accordance with NNR 2023 for both genders.

**Table 3:** Estimated mean micronutrient dietary intake after 4 and 12 weeks on MAD and in the reference population (Norkost 4) for men and women separately, as a percentage of recommendations from the NNR 2023.

Micronutrients	Women			Men			NNR 2023	
	Ref. pop. *n* = 1,049	MAD 4W *n* = 30	MAD 12W *n* = 28	Ref. pop. *n* = 915	MAD 4W *n* = 22	MAD 12W *n* = 16	Recommended/adequate intake men/women	Upper intake limit
Vitamin A, μg RE	↓	✓	✓	✓	✓	✓	800/700	3,000
Vitamin D, μg	↓↓	↓	↓↓	↓↓	✓	✓	10/10	100
Vitamin E, μg	✓	✓	✓	✓	✓	✓	11/10	300
Thiamine, mg	✓	✓	✓	✓	✓	✓	0.1/0.1 /MJ	-
Riboflavin, mg	✓	↓	↓	✓	✓	✓	1.6/1.6	-
Vitamin B6, mg	✓	↓	↓↓	✓	✓	✓	1.8/1.6	25
Vitamin B12, μg	✓	✓	✓	✓	✓	✓	4/4	-
Folate, μg	↓↓	↓	↓↓	↓	↓	↓	330/330	1,000
Vitamin C, mg	✓	↓↓	↓↓	↓	↓↓	↓↓	110/95	1,000
Calcium, mg	↓	↓↓	↓↓	✓	↓	↓	950/950	2,500
Magnesium, mg	✓	↓	↓	✓	↓	↓	350/300	250
Sodium, g	✓	✓	✓	✓	✓	✓	1.5/1.5	2.3
Potassium, g	↓	↓↓	↓↓	✓	↓↓	↓	3.5/3.5	-
Phosphorous, g	✓	✓	✓	✓	✓	↓	0.52/0.52	3.0
Iron, mg	↓↓	↓↓	↓↓↓	✓	✓	✓	9/15	60
Zinc, mg	✓	↓	↓	✓	↓	↓	12.7/9.7	25
Copper, mg	✓	✓	✓	✓	✓	✓	0.9/0.9	5
Iodine, μg	↓	↓↓	↓↓	✓	↓	✓	150/150	600
Selenium, μg	↓↓	↓	↓↓	↓↓	↓	↓	90/75	255

↓: 0–25% lower than recommended intake; ↓↓: 25–50% lower than recommended intake; ↓↓↓: > 50% lower than recommended intake; ✓: According to the recommended intake, Norkost 4 is the reference population. No supplements are included. MAD=modified Atkins diet; SD=standard deviation; NNR 2023=Nordic Nutrition Recommendations 2023; MJ=megajoules; RE=retinol equivalents.

Regarding electrolytes, calcium, magnesium, and potassium, the dietary intakes were lower than the recommended levels for both men and women. Women in the study population ate 1.1 and 1.4 g less potassium than recommended at 4 and 12 weeks, respectively. The mean intake of iron among women was <50% of the recommended 15 mg.

## 4 Discussion

To the best of our knowledge, this is the first study on micronutrient intake in adults on MAD. All reported values are from foods alone, excluding vitamin and mineral supplements.

The intake of fat-soluble vitamins A, D, and E was higher, while the intake of several water-soluble vitamins, minerals, and electrolytes was lower compared with a population-based reference population eating a habitual diet. Higher intake of fat-soluble vitamins was as expected.

With respect to the NNR 2023 recommendations, no nutrients exceeded the upper intake limit, even when adding a multivitamin and multimineral supplement (Nycoplus). However, considerably lower intake (25–50%) was observed in both men and women for vitamin C and potassium. Among women on MAD, vitamin D, folate, calcium, iodine, and selenium were also considerably (25–50%) lower than recommended, while iron at the 12-week follow-up was >50% lower. Furthermore, magnesium and zinc were lower than recommended but less pronounced (<25%) in both men and women. Among women, riboflavin and vitamin B6 were <25% below recommended levels, and among men, folate, calcium, and selenium were <25% below recommended levels.

Low intake of vitamin C is of clinical significance since this vitamin facilitates a wide range of essential functions such as immune response, angiogenesis, neurotransmission, energy metabolism, cell proliferation, and epigenetic regulation, and it protects against oxidative stress (19). Long-term subclinical insufficiency of vitamin C may increase the risk of cardiovascular disease and cancer (19). Of special importance when eating a high-fat diet, carnitine is a semi-essential amino acid needed for transporting fatty acids into mitochondria, and vitamin C is a cofactor in carnitine synthesis.

To the best of our knowledge, low potassium intake was recently, for the first time, reported among children on MAD (9). Considerably lower potassium intake in our adult MAD group compared to both the reference population and recommendations is attributed to restrictions of fruits and vegetables, potatoes, whole-meal bread, and cereals. Ketogenic dietary treatment induces mild metabolic acidosis and may therefore increase the risk of kidney stones (20). To prevent kidney stones, supplementation of potassium citrate is recommended for children on a classical ketogenic diet (15). Notably, multivitamin supplementation products normally do not contain potassium. However, potassium chloride is commercially available in grocery stores as a substitute for salt. Thus, partly substituting sodium chloride with potassium chloride may be a good recommendation for MAD patients, but further investigations are required. For adults on a MAD, the long-term consequences of low potassium intake are unknown but may be unfavorable (21).

Our data show that women fail to reach the recommended daily intake for a greater number of nutrients than men. Due to a smaller body size and lower basal metabolic rate, women need fewer calories than men (22), implying lower intake of micronutrients. Moreover, dietary patterns differ between genders. Vitamin C intake may be higher among women due to women eating more fruits and vegetables than men (23). For many nutrients, dietary reference values are higher for men than for women. However, regarding iron, the estimated requirements are higher for women than men (10).

The Norwegian diet is low in iodine (24), and salt is a minor contributor of iodine in the Norwegian diet (25). Moreover, the lowered salt intake among the participants is as expected since 75% of salt comes from ready-made foods (26). On MAD, the majority of foods are cooked from scratch.

For the clinically important nutrients vitamin D and calcium, the mean intake on MAD among women is lower than recommended. Keeping in mind that the ketogenic diet in children and adults may lead to reduced bone mineralization (27, 28) and anti-seizure drugs may interfere with vitamin D metabolism (29), intake of vitamin D and calcium must be carefully monitored.

The mean fiber intake of the study population (24 g/d) was nearly in accordance with recommendations. This was in line with Tsang et al.’s study (9) and may be due to nutritional advice and homemade bread containing seeds, nuts, and psyllium husk. This contrasts with a recent Swedish study reporting a median fiber intake of 13 g per day among adults eating a low-carbohydrate, high-fat diet not receiving dietary advice (21).

Our study has some limitations. Based on only 12 weeks of intervention, we cannot comment on long-term dietary changes. Moreover, our sample size is small, particularly the male subgroup. The multi-day weighed food record method is considered accurate since all foods are weighed and recorded throughout the day rather than recalled and estimated later. It is commonly used in clinical research (30) and when validating food frequency questionnaires and other dietary intake tools (31, 32). A weakness of using 3 days’ records is that foods with high day-to-day variability can be missed. Therefore, recording more than 3 days could be favorable (33). Values presented are estimations for the group intake and must be interpreted with caution. No adjustment for confounding factors such as age, gender, and BMI was done.

Weighed food records collected over many days impose a high participant burden, which may lead to fatigue and underreporting (31). In this project, we collected two 3-day weighed food records (at 4- and 12-week follow-ups). This approach may achieve higher accuracy than a single recording while not leading to participant non-compliance because of the weeks between the two recordings. A strength is that dietary intake for the two time periods coincides.

Moreover, we speculate that the participant burden may have been lower on a modified Atkins diet than in other studies collecting weighed dietary records (18, 30). During admission, participants were taught how to record food intake, and since the diet was a novel treatment against seizures, participants were highly motivated and were closely followed up during the project period. As part of the protocol, participants weighed all foods eaten throughout the first 4 weeks of dietary treatment.

Differences between the study population and the reference population may lead to bias: The study population was closely monitored within a clinical trial context, while the reference population was contacted via telephone and email. The study group participants suffered from severe epilepsy, while the reference population was sampled to reflect the general Norwegian population. In addition, dietary data were collected using different methods: The reference population completed 24-h dietary recalls and food frequency questionnaires, whereas the study population delivered two 3-day weighed food records.

This study focuses on dietary intake, but it would have been clinically relevant to include selected biochemical endpoints. However, conducting a modified Atkins diet intervention study without supplementation may not be ethically approved. Thus, our participants were supplemented with vitamins and minerals (not included in the reported values), and therefore, biochemical endpoints would not reflect the intake from food alone and are not included.

## 5 Conclusion

In adults with drug-resistant epilepsy, 12 weeks of treatment with MAD was associated with lower mean intakes of several water-soluble vitamins, minerals, and electrolytes compared to recommendations. Notably, the mean estimated intake of vitamin C and potassium was considerably lower than recommended in both men and women. Furthermore, according to our estimations, among women, vitamin D, calcium, iron, iodine, and selenium require attention.

The study highlights the need for systematic dietary monitoring and nutritional advice, including appropriate supplementation during MAD treatment. Our findings support current practice regarding supplementation of vitamins and minerals, but low potassium intake is a novel finding, and this electrolyte is normally not included in supplements. Future studies with longer follow-up are needed to assess the long-term nutritional adequacy and clinical consequences of MAD in adults with epilepsy.

## Data Availability

The raw data supporting the conclusions of this article will be made available by the authors, without undue reservation.
