# Supplementary material for: Micronutrient intake in adults with drug-resistant epilepsy treated with the modified Atkins diet needs monitoring
Source: Front Nutr. 2026 May 7;13:1797011. doi: 10.3389/fnut.2026.1797011 (PMC13190565; doi:10.3389/fnut.2026.1797011)
Supplement: Supplementary file 1 [file Supplementary_File_1.docx]

**Supplement to**

**Micronutrient~~s~~ intake in adults with drug resistant epilepsy treated with modified Atkins diet needs monitoring**

Ida Kjendbakke^1^, Kaja Kristine Selmer^1,4^, Karl Otto Nakken^1^, Dag Hofoss^1^, Per Ole Iversen^2,3^, Magnhild Kverneland^1^

**Affiliations**

^1^National Centre for Epilepsy, Full Member of European Reference Network on Rare and Complex Epilepsies EpiCARE, Oslo University Hospital, Oslo, Norway

^2^Department of Nutrition, University of Oslo, Oslo, Norway

^3^Department of Hematology, Oslo University Hospital, Oslo, Norway

^4^Department of Research and Innovation, Division of Clinical Neuroscience, Oslo University Hospital, Oslo, Norway

**Study population**

Between March 1, 2011, and February 28, 2017, 277 patients from regions across Norway were assessed for eligibility. The study was performed at the National Center for Epilepsy, a tertiary referral center in Norway. It was approved by the Regional Committee for Medical and Health Research Ethics in South-East of Norway (number 2010/2326). Candidates received detailed project information both verbally and in writing. Out of those invited, 88 patients consented to participate. Of these, 56 completed the 4-week dietary treatment, and 45 completed the 12-week follow-up.

The study involved a 12‑week intervention in which participants followed a modified Atkins dietary (MAD) regimen. Before beginning the diet, patients were invited to a brief hospital visit when needed, accompanied by caregivers, to complete baseline assessments and receive guidance. During this visit, they also learned how to record their food intake using weighed dietary records.

The diet was initiated at home on a scheduled start date. The nutritional approach was based on previously published MAD principles, with a daily carbohydrate allowance of no more than 16 grams. Intake of dietary fiber was unrestricted. Participants were encouraged to include generous amounts of high‑fat foods, while neither protein intake nor total energy consumption was restricted. Use of medical nutrition products was generally discouraged.

To support adherence, participants were provided with recipes appropriate for the diet, including options for low‑sugar, high‑fiber baked goods such as breads and crackers. These recipes relied on ingredients like seeds, bran, psyllium husk, nuts, eggs, dairy products, plant oils, and margarine. They also received suggested daily meal plans, which were adjusted for energy needs when relevant. To reduce the likelihood of kidney stone formation, participants were advised to consume 2–3 liters of fluids each day.

The impact of dietary treatment was assessed during single‑day hospital visits scheduled 4 and 12 weeks after the start of the diet. To monitor compliance, ketosis was measured each day using urine test strips (Ketostix, Bayer Healthcare, Leverkusen, Germany). Participants tested both their first morning urine sample and another sample collected before their final meal of the day. During the follow‑up visits at weeks 4 and 12 participants also completed three‑day weighed food records. These logs were reviewed in consultations with the dietitian (MK) to provide tailored dietary guidance.

Eligible patients were >16 years; had diagnosed epilepsy according to the International League Against Epilepsy’s classification (1); had at least 3 seizures per month, having tried at least 3 AEDs, including current treatment; a body mass index >18.5 kg/m2; motivated for and capable of adhering MAD, and if required, with assistance. Exclusion criteria are described elsewhere (2).

Reasons for non-participation included inability to prepare or eat the restrictive diet, changes in medication, changes in diagnosis, and newly emerged disease incompatible with ketogenic dietary treatment. Reasons for discontinuation included increased seizure frequency, allergic reactions, food intolerance, pregnancy, and loss of motivation. **Figure S-1** provides an overview of the study population and the inclusion and exclusion criteria. Of the 45 who completed the 12-week dietary treatment, 4 failed to deliver the 3-day food record at the 4-week follow-up.


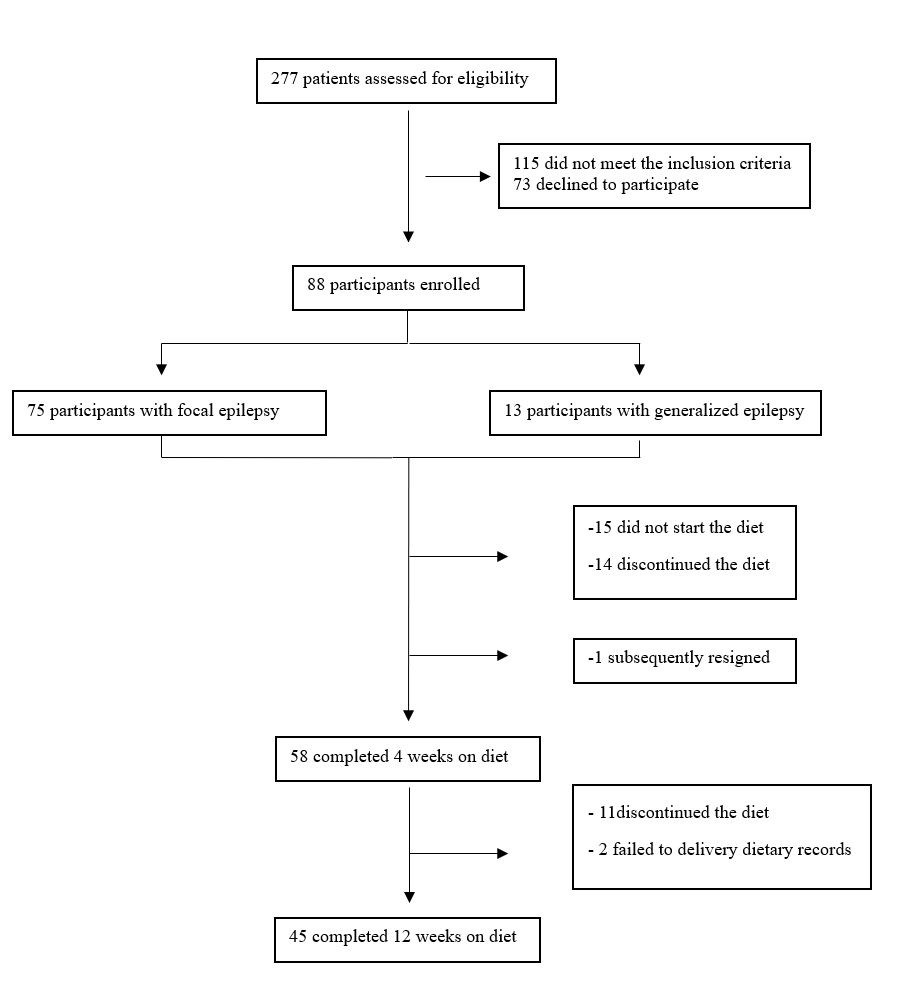


**Figure S1.** Flow chart of the study population.

**Supplement Table S1A** Estimated mean micronutrient dietary intake, supplements not included, after 4 and 12 weeks on MAD for **women**, compared to the women of Norkost 4 reference population. Bold font indicates a value below NNR2023 recommendation.

|  | Norkost4 | 4 weeks on MAD  Mean (SD)  n = 30 | Comparison 4 weeks MAD to Norkost4 | | 12 weeks on MAD  Mean (SD)  (n = 28-29) | Comparison 12 weeks MAD to Norkost4 | | NNR2023 | |
| --- | --- | --- | --- | --- | --- | --- | --- | --- | --- |
|  | Mean (SD)  n = 1049 |  | Mean difference (SD) | p |  | Mean difference (SD) | p | RI or AI women >18 yrs | UL |
| Vitamin A, µg RE | **638 (671)** | 786 (318) ^*^ | 147 (324) | 0.019 | 756 (368) ^*^ | 118 (376) | 0.108 | 700 | 3000 |
| Vitamin D, µg | **5 (5)** | **9 (6)** | 4 (6) | <0.001 | **7.5 (4.7)** | 3 (4.8) | <0.001 | 10 | 100 |
| Vitamin E, µg | 13 (7) | 23.9 (11) | 11 (12) | <0.001 | 23 (14) | 10 (14) | <0.001 | 10 | 300 |
| Thiamine, mg | 1.5 (0.8) | 1.2 (0.5) | -0.3 (0.6) | 0.011 | 1.0 (0.4) | -0.5 (0.4) | <0.001 | 0.1 MJ | - |
| Riboflavin, mg | 1.6 (0.8) | **1.4 (0.6)** | -0.2 (0.7) | 0.061 | **1.3 (0.6)** | -0.3 (0.6) | 0.010 | 1.6 | - |
| Vitamin B6, mg | 1.6 (0.9) | **1.4 (0.5)** | -0.2 (0.5) | 0.010 | **1.2 (0.4)** | -0.4 (0.4) | <0.001 | 1.6 | 25 |
| Vitamin B12, µg | 5.4 (4.9) | 7.6 (12.6) | 2.2 (13.1) | 0.336 | 6.8 (10.6) | 1.4 (10.9) | 0.486 | 4 | - |
| Folate, µg | **235 (114)** | **251 (114)** | 16 (119) | 0.452 | **225 (96)** | -10 (99) | 0.591 | 330 | 1000**^2^** |
| Vitamin C, mg | 95 (82) | **58 (32)** | -38 (33) | <0.001 | **50 (29)** | -45 (30) | <0.001 | 95 | 1000 |
| Calcium, mg | **857 (493)** | **582 (278)** | -275 (290) | <0.001 | **524 (234)** | -333 (240) | <0.001 | 950 | 2500 |
| Magnesium, mg | 312 (130) | **279 (127)** | -33 (133) | 0.171 | **247 (130)** | -65(133) | 0.012 | 300 | 250**^3^** |
| Sodium, g | 2.6 (1.4) | 2.0 (0.9) | -0.6 (1.0) | 0.001 | 1.8 (0.9) | -0.8 (1.0) | <0.001 | 1.5 | 2.3 |
| Potassium, g | **3.3 (1.3)** | **2.4 (0.8)** | -0.9 (0.8) | <0.001 | **2.1 (0.7)** | -1.2 (0.7) | <0.001 | 3.5 | - |
| Phosphorous, g | 1.5 (0.6) | 1.3 (0.4) | -0.2 (0.5) | 0.044 | 1.1 (0.4) | -0.3 (0.4) | <0.001 | 0.52 | 3.0 |
| Iron, mg | **9 (4)** | **8 (3)** | -0.7 (4) | 0.298 | **7 (3)** | -2 (3) | 0.003 | 15 | 60 |
| Zinc, mg | 9.7 (4.7) | **9.6 (3.7)** | -0.06 (3.9) | 0.934 | **8.4 (3.7)** | -1.3 (3.8) | 0.073 | 9.7 | 25 |
| Copper, mg | 1.2 (0.6) | 1.3 (1.2) | 0.1 (1.2) | 0.606 | 1.1 (1.1) | -0.1 (1.1) | 0.759 | 0.9 | 5 |
| Iodine, µg | **144 (190)** | **100 (57)** | -44 (59) | <0.001 | **99 (61)** | -46 (62) | <0.001 | 150 | 600 |
| Selenium, µg | **46 (41)** | **61 (21)** | 15 (22) | <0.001 | **56 (23)** | 10 (24) | 0.034 | 75 | 255 |

*n=29/27 one outlier removed, **^2,3^** applies to intake from supplements. Norkost4 is the reference population. No supplements are included. MAD = Modified Atkins Diet; SD=standard deviation; NNR2023 = Nordic Nutrition Recommendations 2023; RE=Retinol Equivalents; UL=upper intake level

**Supplement Table S1B.** Estimated mean micronutrient dietary intake at 4 and 12 weeks on MAD for **men**, compared to male Norkost4 reference population. Bold font indicates a value below NNR2023 recommendation.

|  | Norkost4 | 4 weeks on MAD  Mean (SD)  n = 22 | Comparison 4 weeks MAD to Norkost4 | | 12 weeks on MAD  Mean (SD)  (n = 16) | Comparison 12 weeks MAD to Norkost4 | | NNR2023 | |
| --- | --- | --- | --- | --- | --- | --- | --- | --- | --- |
|  | Mean (SD)  n = 915 |  | Mean difference (SD) | p |  | Mean difference (SD) | P | RI or AI  men >18 yrs | UL |
| Vitamin A, µg RE | 822 (918) | 976 (396) | 153 (396) | 0.083 | 1054 (542) | 231 (543) | 0.108 | 800 | 3000 |
| Vitamin D, µg | **7 (8)** | 12 (7) | 5 (7) | 0.002 | 14 (10) | 7 (11) | 0.014 | 10 | 100 |
| Vitamin E, µg | 17 (9) | 31 (10) | 14 (11) | <0.001 | 31 (12) | 14 (15) | <0.001 | 11 | 300 |
| Thiamine, mg | 1.9 (1.0) | 1.5 (0.5) | -0.4 (0.6) | 0.001 | 1.5 (0.7) | -0.4 (0.9) | 0.043 | 0.1 MJ | - |
| Riboflavin, mg | 2.3 (1.3) | 1.6 (0.6) | -0.7 (0.6) | <0.001 | 1.8 (1.0) | -0.5 (1.3) | 0.043 | 1.6 | - |
| Vitamin B6, mg | 2.1 (1.1) | 1.7 (0.6) | -0.4 (0.6) | 0.004 | 1.9 (0.9) | -0.2 (1.1) | 0.490 | 1.8 | 25 |
| Vitamin B12, µg | 7.9 (6.8) | 7.9 (3.3) | -0.05 (3.5) | 0.943 | 8.1 (5.5) | 0.2 (6.9) | 0.903 | 4 | - |
| Folate, µg | **279 (132)** | **265 (95)** | -14 (101) | 0.487 | **295 (132)** | 16 (167) | 0.626 | 330 | 1000**^2^** |
| Vitamin C, mg | **98 (81)** | **68 (42)** | -30 (45) | 0.003 | **79 (56)** | -19 (71) | 0.202 | 110 | 1000 |
| Calcium, mg | 1085 (681) | **737 (371)** | -348 (393) | <0.001 | **840 (705)** | -244 (900) | 0.185 | 950 | 2500 |
| Magnesium, mg | 389 (160) | **306 (107)** | -83 (114) | 0.002 | **315 (128)** | -74 (163) | 0.018 | 350 | 250**^3^** |
| Sodium, g | 3.6 (1.9) | 2.2 (1.0) | -1.3 (1.0) | <0.001 | 2.3 (0.9) | -1.3 (1.2) | <0.001 | 1.5 | 2.3 |
| Potassium, g | 4.2 (1.6) | **2.6 (0.8)** | -1.6 (0.8) | <0.001 | **3.1 (1.1)** | -1.1 (1.4) | 0.001 | 3.5 | - |
| Phosphorous, g | 2.0 (0.8) | 1.6 (0.8) | -0.4 (0.5) | <0.001 | 1.6 (0.7) | -0.3 (0.9) | 0.075 | 0.52 | 3.0 |
| Iron, mg | 12 (5) | 9.3 (3.9) | -2.7 (4.1) | 0.004 | 10 (7) | -2.2 (8) | 0.190 | 9 | 60 |
| Zinc, mg | 13.3 (6.5) | **11.3 (4.1)** | -2.0 (4.4) | 0.036 | **11.6 (6.2)** | -1.7 (7.9) | 0.300 | 12.7 | 25 |
| Copper, mg | 1.4 (0.6) | 1.1 (0.4) | -0.3 (0.5) | 0.005 | 1.1 (0.6) | -0.3 (0.7) | 0.049 | 0.9 | 5 |
| Iodine, µg | 175 (217) | **145 (115)** | -30 (122) | 0.237 | 193 (164) | 18 (209) | 0.627 | 150 | 600 |
| Selenium, µg | **65 (43)** | **77 (29)** | 17 (30) | 0.010 | **84 (42)** | 24 (54) | 0.036 | 90 | 255 |

**^2,3^** applies to intake from supplements. Norkost4 is the reference population. No supplements are included. MAD = Modified Atkins Diet; SD=standard deviation; NNR2023 = Nordic Nutrition Recommendations 2023; RE=Retinol Equivalents; UL=upper intake level

**Supplement Table S2.** Min and max intake of micronutrients in men and women on MAD. Bold font indicates excessive intake.

|  | Women | | Men | | NNR2023 | |
| --- | --- | --- | --- | --- | --- | --- |
|  | 4 weeks  n=30 | 12 weeks  n=28/29** | 4 weeks  n=22 | 12 weeks  n=16 | Recommended/ adequate Intake Men/ Women | UL |
| Vitamin A, RE | 283 – 1542* | 295 – 1755* | 284 – 1750 | 234-2294 | 800 / 700 | 3000 |
| Vitamin D, µg | 2.4 – 25.7 | 0.3 – 21.8 | 3.5 – 34.7 | 3.9 – 42.5 | 10 / 10 | 100 |
| Vitamin E, µg | 8.8 – 57.3 | 1.7 – 62.8 | 10.5 – 50.9 | 16.2 – 56.5 | 11 / 10 | 300 |
| Thiamine, mg | 0.4 – 2.3 | 0.4 – 2.0 | 0.4 – 2.5 | 0.4 – 3.0 | 0.1 / 0.1 /MJ | - |
| Riboflavin, mg | 0.6 – 3.9 | 0.3 – 3.7 | 0.6 – 2.8 | 0.5 – 4.0 | 1.6 / 1.6 | - |
| Vitamin B6, mg | 0.7 – 2.6 | 0.5 – 2.0 | 0.6 – 2.6 | 0.7 – 4.1 | 1.8 / 1.6 | 25 |
| Vitamin B12, µg | 1.1 – 73.0 | 0.7 – 61.1 | 4.0 – 15.1 | 2.6 – 23.4 | 4 / 4 | - |
| Folate, µg | 97.5 - 627 | 42.3 – 522.4 | 93 – 554 | 142 – 594 | 330 / 330 | 1000^2^ |
| Vitamin C, mg | 11.1 – 160.0 | 8.6 – 124.4 | 3 - 157 | 6 – 228 | 110 / 95 | 1000 |
| Calcium, mg | 198 - 1445 | 149 – 1124 | 235 - 1450 | 176 – **2760** | 950 / 950 | 2500 |
| Magnesium, mg | 103 - 598 | 52 – 671 | 129 - 507 | 173 – 573 | 350 / 300 | 250^3^ |
| Sodium, g | 0.3 – **5.7** | 0.4 – **4.3** | 0.7 – **5.7** | 0.9 – **4.1** | 1.5 /1.5 | 2.3 |
| Potassium, g | 1.0 – 4.0 | 0.5 – 3.7 | 1.0 – 4.2 | 1.2 – 5.1 | 3.5 / 3.5 | - |
| Phosphorous, g | 0.73 – 2.3 | 0.36 – 2.02 | 0.68 – 2.44 | 0.67 – 3.03 | 0.52 / 0.52 | 3.0 |
| Iron, mg | 3 – 16 | 1 – 14 | 5 - 21 | 3 – 31 | 9 / 15 | 60 |
| Zinc, mg | 3.7 – **18.6** | 2.0 – **16.4** | 4.8 – **18.9** | 3.1 – **24.3** | 12.7 / 9.7 | 25 |
| Copper, mg | 0.3 – 6.8 | 0.1 – 6.1 | 0.5 – 2.2 | 0.5 – 2.5 | 0.9 / 0.9 | 5 |
| Iodine, µg | 28.8 – 318.7 | 4.4 – 261.9 | 30 - 432 | 43 - **725** | 150 / 150 | 600 |
| Selenium, µg | 25.7 - 119 | 11.3 – 124.1 | 32 - 126 | 30 – 172 | 90 / 75 | 255 |

*n=29/27 one outlier removed. ** variation in n due to missing values. **^2,3^** applies to intake from supplements. No supplements are included. MAD = Modified Atkins Diet; SD=standard deviation; NNR2023 = Nordic Nutrition Recommendations 2023; RE=Retinol Equivalents; UL=upper intake level

1. Scheffer IE, Berkovic S, Capovilla G, Connolly MB, French J, Guilhoto L, et al. ILAE classification of the epilepsies: Position paper of the ILAE Commission for Classification and Terminology. Epilepsia. 2017;58(4):512–21.

2. Kverneland M, Molteberg E, Iversen PO, Veierod MB, Tauboll E, Selmer KK, et al. Effect of modified Atkins diet in adults with drug-resistant focal epilepsy: A randomized clinical trial. Epilepsia. 2018;59(8):1567–76.
